# A Comparison of Sleep Duration Accuracy Between Questionnaire and Accelerometer in Middle Childhood

**DOI:** 10.7759/cureus.47236

**Published:** 2023-10-17

**Authors:** Kanae Kanda, Tomohiro Hirao, Nlandu R Ngatu, Akitsu Murakami, Yusuke Yamadori, Katsunori Yokoyama, Yoichi Hoshikawa, Tetsuo Minamino

**Affiliations:** 1 Department of Public Health, Faculty of Medicine, Kagawa University, Miki, JPN; 2 Department of Palliative Care, Faculty of Medicine, Kagawa University, Miki, JPN; 3 Department of Anesthesiology, Takamatsu Red Cross Hospital, Takamatsu, JPN; 4 Department of Health and Welfare, Kagawa Prefecture Tosan Health and Welfare Office, Takamatsu, JPN; 5 Department of Health and Welfare, Kagawa Prefectural Government Office, Takamatsu, JPN; 6 Department of Cardiorenal and Cerebrovascular Medicine, Faculty of Medicine, Kagawa University, Miki, JPN

**Keywords:** sleep duration, questionnaire, physical activity, middle childhood, children, accelerometer

## Abstract

Purpose

Healthy sleep is vital to children’s well-being, and assessing sleep efficiently and accurately can help understand children's lifestyles. Due to the difficulty in objectively measuring sleep duration using wearable sensors in large-scale surveys of children, self-administered questionnaires are often used in Japan; however, their accuracy is uncertain. We evaluated and compared the accuracy of questionnaire-based sleep times to those of wearable sensors.

Methods

This observational study was conducted between November 2019 and January 2020. A self-administered questionnaire on lifestyle habits and ActiGraph GT3X+ (ActiGraph, Inc., Pensacola, USA) accelerometer data were collected from 40 fourth-grade elementary school students in Kagawa Prefecture, Japan. We analyzed measurements for 256 days out of 280 days (40 persons × 7 days) after excluding days when the rate of wearing the accelerometer was < 90%.

Results

The median sleep duration per accelerometry was 453 minutes, and the median time in bed was 519 minutes. Questionnaire-based time in bed was 11 minutes longer, with relatively high inter-individual variability. The difference in bedtime was 26 minutes earlier, and wake-up time was 12 minutes earlier for the questionnaire. The average sleep efficiency was 87.4%, and one-third of the children had sleep efficiency < 85%.

Conclusion

The difference in sleep duration by questionnaire compared to accelerometry was approximately 10 minutes, suggesting the questionnaire may determine sleep duration with accuracy.

## Introduction

It has long been reported that Japanese adults and children sleep the shortest hours in the world [1]. In a survey of Japanese children's life rhythm over time elementary through high school students have been going to bed later and sleeping shorter hours [2]. The average sleep time of children from first grade to high school tends to get shorter every year, from 9 h 15 min in the first grade to 6 h 30 min in high school.

Epidemiological studies have reported an association between short sleep duration and the development of obesity [3]. In school-aged children, a disrupted lifestyle due to nighttime activity, sedentary activities, and decreased physical activity (PA) are associated with the onset of obesity [4,5]. Sleeping less than 8 h per day increases the risk of developing obesity 2.87 times, and longer screen times devoted to gaming and other activities are associated with a higher prevalence of obesity [6].

These current conditions have led to the formulation of guidelines by relevant academic societies in Japan for the prevention of obesity starting in childhood. For example, the treatment guidelines for childhood obesity recommend that school-aged children should avoid sleeping less than 8 h per day and long periods of screen time [7].

Local governments, local medical associations, boards of education, and schools are collaborating to provide checkups for children to screen for health conditions, aiming to prevent lifestyle-related diseases [8]. For children and guardians who require improvement for obesity, and have abnormal blood test results (elevated hemoglobin A1c, elevated triglycerides, low high-density lipoprotein (HDL) cholesterol) or lifestyle-related diseases, lifestyle guidance is provided with follow-up observation and a visit to a medical institution is recommended. This pediatric lifestyle-related disease prevention checkup also includes a survey on diet, sleeping time, and exercise time.

Of concern in this survey was sleep time measurement accuracy. Sleep time can be accurately measured by polysomnogram (PSG) [9] or accelerometer. Accelerometers are small devices worn on the wrist that can measure acceleration in three dimensions and can analyze sleep, as well as PA [10]. In recent health research studies, accelerometers made by ActiGraph, Inc., have been widely used because of their high accuracy and reliability [11]. Moreover, sleep measurement using this accelerometer has been reported to be as sensitive as a PSG in school-aged children [12,13]. Since it is difficult and impractical to have many children wear accelerometers at one time in situations such as health checkups, surveys are commonly conducted. The questionnaire is intended to allow children to reflect on their daily habits by completing the questionnaire themselves. However, the accuracy of the sleeping hours on the questionnaires answered by the children themselves has not been verified to date. Therefore, the purpose of this study was to verify the accuracy of sleep duration obtained from a questionnaire by distributing a self-administered questionnaire and an accelerometer, the "ActiGraph GT3X+" (ActiGraph, Inc., Pensacola, USA) to fourth-grade elementary school students.

## Materials and methods

Participants and procedures

The study used an observational research design with children. The study participants were fourth-grade elementary school students aged 9-10 years living in Kagawa Prefecture, Japan. The participating children and their parents or guardians were informed of the risks and benefits of this study, and written informed consent was provided by a parent or guardian for each participant. Those who did not provide consent and children who refused to wear the accelerometers were excluded. Participants were kept anonymous with a research ID attached so that the authors could not identify individual participants. The study was conducted from November 2019 through January 2020. The study period did not include the holiday seasons.

Measures

Sleep Duration

Sleep duration was evaluated using a triaxial accelerometer (ActiGraph GT3X+) [10]. Children were instructed to wear the accelerometer on their non-dominant hand 24 h/day for seven consecutive days and to remove it only when bathing, showering, or swimming. The wristband type is more practical and comfortable than a hip-worn accelerometer [14] and is reportedly reliable and valid for measuring overall PA in children [15].

The following sleep analysis parameters were measured: bedtime and wake time, time in bed (TIB), sleep onset latency (SOL), total sleep time (TST), waking after sleep onset (WASO), frequency of the average duration of waking periods, total amount of body movement, and sleep efficiency (SE). The Cole-Kripke algorithm [16] was used; sleep and wakefulness were determined using 60-s epochs (time blocks). In using the GT3X+, we decided to apply the Cole-Kripke algorithm for sleep analysis, which was validated in a sample of elementary school students to be more sensitive to sleep detection than the Sadeh algorithm [17]. Sleep duration included only nocturnal sleep. Bedtime and wake time were determined manually by examining individual actograms. Manual scoring has been found to correlate more strongly with PSG readings than computer-based automated analysis [18].

TIB and TST are calculated by body movements during sleep. SE refers to the ratio of time spent asleep to TIB and is calculated as follows: TST/TIB x 100 (%). The higher the SE value, the better the sleep quality. The cutoff value was 85% SE, which is the standard for determining good and bad SE [19]. The measured sleep analysis parameters, which have been described elsewhere in the literature [20], were defined as follows:

a) Sleep timing:

Bedtime: time the participant goes to bed.

Wake time: time the participant gets out of bed.

Sleep onset: clock time of the first consecutive minute scored as evening sleep.

Sleep offset: clock time of the first consecutive minute scored as morning waking time.

b) Sleep quantity:

SOL: time between bedtime and sleep onset.

Sleep period time (SPT): time elapsed between sleep onset and offset (excludes SOL).

WASO: number of minutes scored as awake between sleep onset and offset.

TST: represents true sleep time calculated as SPT minus WASO.

TIB: time elapsed from getting into bed to getting out of bed (includes SOL).

c) Sleep quality:

SE: percent of time spent asleep between sleep onset and offset. This measure excludes sleep latency. SE = TST/SPT or TST/TIB. This study used the latter definition of SE (TST/TIB).

Physical activity

PA, number of steps, activity intensity, and activity duration, were measured using the ActiGraph GT3X-BT. Using Freedson’s cut points for children [21,22], the measured PA was divided into four levels: sedentary, light, moderate, and vigorous. We note that actigraphy uses an inclinometer (body position analysis) to determine whether the subject is in a standing, sitting, or lying position. Since sedentary behavior determined by actigraphy also includes TIB, in this study, the duration of sedentary activity minus TIB was defined as “sedentary time.”

Questionnaire

At the beginning of this project, a form to record data for seven days was distributed and the children were instructed to complete this questionnaire themselves each day. Participants were advised to fill out the form by the end of the day so that their memory of that day would be fresh. We advised parents to help their children complete the daily sleep questionnaires, if needed. The accelerometers and questionnaire forms were collected after one week. The questionnaire comprised the following four questions:

“At what time did you go to bed yesterday and at what time did you get up this morning?”

“Please tell us how long you spent walking to school today.”

“Did you go to a sports club or swimming today?”

“How many hours did you spend looking at game consoles, smartphones, televisions, tablets, computers, or other screens today?”

The primary outcome measures evaluated in this study were sleep duration by accelerometry and the degree of accuracy (error and error distribution) of sleep duration assessed by the questionnaire. Secondary outcome measures were the associations between sleep duration, number of steps, and sitting time.

Sample size estimation

In a previous study on sleep in children [23], the average sleep duration for children was 522 min (8 h 42 min), with a standard deviation of 30 min. Assuming an error of ± 10 min and a reliability of 95%, the required number of participants was calculated as 34. Considering possible study dropouts and the number of children per class, the required number of participants was set at 40.

Ethical considerations

The study was conducted according to the guidelines of the Declaration of Helsinki and was approved by the ethics committee of the Kagawa University School of Medicine (approval no.: 2019-141; approval date: July 29, 2019). The benefits and risks of the study were explained to the participants using an explanatory document. After ensuring that the participants fully understood this explanation, their free and voluntary informed consent to participate in this study was obtained in writing. Since this study was conducted with children, the children’s parents/guardians were regarded as legal representatives and their consent for their children to participate in the study was obtained as well. To ensure the participants fully understood the study items were explained as clearly as possible.

Statistical analyses

All data measured with the wearable accelerometer were analyzed using ActiLife software (v.6.13.4, ActiGraph, Inc., Pensacola, USA). The duration of wear per day was calculated. The days with a wearing rate < 90% were excluded from the analysis. The measurement error for sleep duration assessed by questionnaire and on accelerometry was determined, and differences were tested by t-test. Pearson’s correlation coefficient analysis was used to evaluate the associations between sleep duration and some lifestyle-related factors. A p-value of < 0.05 was considered statistically significant. JMP Pro 16.1 statistical software (SAS Institute, Cary, NC, USA) was used for statistical processing.

## Results

Table 1 provides the characteristics of the analytic sample. Descriptive statistics were presented primarily as medians and interquartile ranges, per day basis. One participant was excluded due to refusal to wear the accelerometer. In total, 40 participants, with a mean age of 9.7 (0.5) years, 26 boys (65.0%) and 14 girls (35.0%), were in the final analysis. All participants were Japanese citizens. The median body mass index (BMI) was 17.1 (interquartile range [IQR], 16.1-18.4) and was relatively higher in girls than in boys: 18 (IQR, 16.5-18.4) vs. 16.7 (IQR, 16-18). We evaluated results for the measurements of a total of 280 days (40 participants × 7 days); 256 days were included in the analyses because we excluded days when the rate of wearing the accelerometer was < 90%.

**Table 1:** Participant descriptive characteristics Participants, n = 40; samples analyzed, 256 days. SD: standard deviation; IQR: interquartile range; BMI: body mass index

Parameter	All (n = 40)		Boys (n = 26)		Girls (n = 14)	
	Median	(IQR)	Median	(IQR)	Median	(IQR)
Height (cm)	136	(131-141)	136	(132-140)	136.2	(131-146.2)
Weight (kg)	31	(29.7-34)	31	(29-32.3)	33	(30.2-35)
BMI (kg/m^2^)	17.1	(16.1-18.4)	16.7	(16-18)	18	(16.5-18.4)

Table 2 shows the aggregate results for the accuracy of sleep duration. The sleep outcome variables were bedtime, wake time, TIB, TST, and SE. We noted that 53.1% of all participants went to bed between 21:30 and 22:00 and that the difference in bedtime was 26 min earlier using the questionnaire (p < .0001). In addition, 53.9% of all participants woke up before 7:00 a.m., with 6:30 a.m. being the most common wake-up time. The difference in wake-up time was 12 min earlier for the questionnaire (p < .0001). The median TIB (the time spent in bed) was 519 min (IQR, 489-547) by accelerometry and 530 min (IQR, 507-559) by the questionnaire. The TIB measurement was 11 min longer (IQR, -19 to -41 min, p = .015) when using the questionnaire. The median TST (the time spent sleeping), as measured by the accelerometer, was 453 min (IQR, 416-489). SE was 87.4%, and one-third of all participants had a SE < 85%.

**Table 2:** Sleep time from the questionnaire survey versus accelerometer Participants, n = 40; samples analyzed, 256 days. IQR: interquartile range; TIB: time in bed (from the time into bed to the time out of bed); SE: sleep efficiency, the percent of time asleep between sleep onset and offset excluding sleep latency; SE=TST/TIB (includes sleep onset latency); sedentary time, static sitting or standing activity; screen time, using items such as games, smartphones, personal computers, tablets, televisions.

Variables	Questionnaire survey		ActiGraph GT3X+		Difference		
	Median	(IQR)	Median	(IQR)	Median	(IQR)	p-value
In bed	21:50	(21:30-22:08)	22:00	(21:34-22:22)	0:26	(0:14-0:46)	< 0.0001
Out bed	6:30	(6:20-7:00)	6:33	(6:16-7:03)	0:12	(0:05-0:34)	< 0.0001
TIB (min)	530	(507-559)	519	(489-547)	11	(-19-41)	0.0015
TST (min)	---	---	453	(416-489)	---	---	---
SE (%)	---	---	88	(83-92)	---	---	---
Sedentary time (min)	---	---	244	(181-332)	---	---	---
Number of steps	---	---	14971	(12344-17176)	---	---	---
Walking time to and from school (min)	12	(0-30)	---	---	---	---	---
Availability of sports club activities (yes, %)	40	(15.6%)	---	---	---	---	---
Screen time (min)	90	(50-150)	---	---	---	---	---

For PA per day as measured by accelerometry, the median sedentary time was 699 min (IQR, 646-790). Median sedentary time calculated by subtracting TIB from total sedentary time was 244 min (IQR, 181-332). The median number of steps taken was 14,971. When compared to the time spent walking as derived from the questionnaire, we found that most steps were taken on weekdays during a school day (between home and school). The median screen time as assessed by the questionnaire was 90 min (IQR, 50-150); 41.4% of the participants had more than 2 h per day of screen time. Only 15.6% of the total participants answered that they engaged in sports activities (sports clubs or swimming) on the day of the survey. Twenty-four (60%) of the respondents did not exercise regularly on any day of the week.

As a secondary analysis, we analyzed associations between sleep duration and lifestyle-related factors. A single correlation analysis using Pearson's correlation coefficients showed a weak negative correlation between sleep duration and sedentary time. However, the correlation coefficient was low and did not provide definitive evidence of an association (r = -0.249, p < 0.0001) (Table 3).

**Table 3:** Relationships between total sleep time and lifestyle factors N = 40; samples analyzed, 256 days. Shown are the r values of Pearson’s correlation coefficient with accompanying p values.

Covariate	r	p
Body mass index (kg/m^2^)	0.058	0.3617
In bed	-0.041	0.5164
Out of bed	0.162	0.0098
Sedentary time	-0.249	<0.0001
Number of steps	-0.038	0.5420
Availability of sports activities	0.031	0.6216
Screen time	-0.069	0.2762

## Discussion

This study examined the difference between sleep duration measured objectively by accelerometry and subjectively using a questionnaire. We also evaluated whether lifestyle-related factors, such as the number of steps taken per day and the sedentary time, were associated with sleep duration. To accomplish these aims, we measured sleep duration for one week in children aged 9-10 years using accelerometry and our study questionnaire, within a community-based study. The difference between sleep duration in children who responded to the questionnaire and the objective time measured by accelerometry was approximately 10 min, suggesting that the questionnaire provided an accurate determination of sleep duration.

Objective measurements using accelerometry or PSG are difficult to obtain with large samples, especially when measuring data for children. One small study compared diary entries to accelerometer estimates and although the bedtime differed by approximately 20 min, the TST differed by only 0.7 min, demonstrating a strong correlation (r = 0.93) between the two modalities [24]. This result suggests that a questionnaire can provide reasonably accurate results within pediatric research. However, since differences between estimates derived from accelerometry and questionnaires vary from individual to individual, it is necessary to devise questions that lead to the best accuracy. It is important to note that there is a difference between the time one gets into bed and the time of actual sleep onset [25]. Since the questionnaire allows the individual to decide which time will be considered the beginning of sleep, measurement errors are likely to occur. Moreover, when using a questionnaire, responses are affected by recall bias [26]. Therefore, it is necessary to answer the day’s questions on the same day or as close to that day as possible so that the respondent’s memory will be clear.

SE was used to measure sleep quality. Although no baseline for SE has been established, a recent report of polysomnographic parameters scored using the American Academy of Sleep Medicine criteria showed a SE of 85.7% for the total sample [27]; the average SE in middle childhood (5-12 years) was 89.6% using the same ActiGraph model as in our study [13]. For the children in our sample, the average was 87.4%, similar to previous studies. A concern was that approximately one-third of the children in the study sample had an SE of < 85%, suggesting that they may either have been unable to fall asleep after going to bed or may have been engaged in another activity before going to sleep. TIB, which is used to calculate SE, includes time spent engaging in non-sleep-related activities, such as reading, watching television, or lying in bed and thinking about the next day; this can distort SE measurements if taken literally [25]. These results suggest that when asked about sleep duration in a questionnaire, some individuals recorded the time they went to bed and others recorded the time they think they fell asleep. Therefore, our suggestion is that when asking about sleep duration in a questionnaire, it is necessary to provide a definition of sleep to the subjects in advance to avoid differences.

Regarding the association between sleep duration and lifestyle-related factors, we found evidence of an association only with sedentary time; no associations with other factors. Previous studies using actigraphy found that children engage in much sedentary behavior and little moderate-to-vigorous PA at school [28]. Moreover, a higher proportion of the school day spent in %MVPA relative to %sedentary times and %LPA was significantly associated with higher tests for gross motor development total scores [29]. In terms of mental health, shorter sleep duration in children has been shown to increase the risk of symptoms of emotional and behavioral disorders measured two years later [30]. Thus sleep is closely related to the amount of PA and to sedentary behavior, which are essential items when assessing lifestyle and health behaviors in children. In this study, we believe the reason sedentary activity was moderately associated and not PA, despite some children taking part in sports activities, was due to the small percentage of children involved in sports activities, and the effect of longer sitting time at school was more significant.

Strengths and limitations

The current observational study of children investigated the accuracy of sleep measured objectively using accelerometry and subjectively using an epidemiological survey questionnaire. This is a substantial strength of this research because this comparison has not been previously clarified. However, our results should be considered in relation to the limitations of the present research.

First, this study was limited by its small sample size. Since the study was conducted within a small population sample in one community in Japan, generalizations should be made with caution. Similarly, the children enrolled in this study were limited to fourth graders, and the results regarding their lifestyle and routines cannot be applied to all elementary school children. Second, because the period for measurement was one week, only short-term and transitory results may have been detected. Since we analyzed the data for one aggregated week, we were unable to examine the characteristics of days of the week or of weekdays and holidays in detail. Third, although SE could be calculated from accelerometers, we were not able to determine the factors that affect SE. Finally, because the purpose of this study was to examine sleep duration in detail and this was an observational study, we did not examine the effects of short sleep durations or late bedtimes on lifestyle.

## Conclusions

The results of this observational study indicated that, in reference to sleep parameters at ages 9-10 years, questionnaire measurements may be comparable to those derived from accelerometry. This suggests that a better, evidence-based questionnaire design will lead to a more accurate understanding of sleep duration. These findings provide implications for public health interventions and preventive medical initiatives.

## References

[1] Mindell JA, Sadeh A, Wiegand B, How TH, Goh DY (2010). Cross-cultural differences in infant and toddler sleep. Sleep Med.

[2] Japan Society of School Health (2023). Japan Society of School Health. Report on the 2018-2019 school year health status surveillance project for children (in Japanese). https://www.gakkohoken.jp/book/ebook/ebook_R010120/index_h5.html#1.

[3] Felső R, Lohner S, Hollódy K, Erhardt É, Molnár D (2017). Relationship between sleep duration and childhood obesity: systematic review including the potential underlying mechanisms. Nutr Metab Cardiovasc Dis.

[4] Sekine M, Yamagami T, Handa K (2002). A dose-response relationship between short sleeping hours and childhood obesity: results of the Toyama Birth Cohort Study. Child Care Health Dev.

[5] Anderson SE, Andridge R, Whitaker RC (2016). Bedtime in preschool-aged children and risk for adolescent obesity. J Pediatr.

[6] Anderson SE, Whitaker RC (2010). Household routines and obesity in US preschool-aged children. Pediatrics.

[7] Japan Society for the Study of Obesity (2017). Guidelines for the Treatment of Childhood Obesity (in Japanese). Tokyo: Life Science.

[8] Kagawa Prefecture Health and Welfare Department (2023). Kagawa Prefecture Health and Welfare Department. Health and Welfare General Affairs Division. Pediatric Lifestyle-Related Disease Prevention Medical Checkup Manual. March.

[9] Rundo JV, Downey R 3rd (2019). Polysomnography. Handb Clin Neurol.

[10] Baley G, Wyatt J (2018). Actigraphy Monitoring Wearables in Clinical Trials: Overcoming Barriers to Adoption & Strategies for Successful Technology Implementation. https://landing.theactigraph.com/overcoming-barriers-to-adoption-and-strategies-for-successful-technology-implementation.

[11] Migueles JH, Cadenas-Sanchez C, Ekelund U (2017). Accelerometer data collection and processing criteria to assess physical activity and other outcomes: a systematic review and practical considerations. Sports Med.

[12] Meltzer LJ, Montgomery-Downs HE, Insana SP, Walsh CM (2012). Use of actigraphy for assessment in pediatric sleep research. Sleep Med Rev.

[13] Meltzer LJ, Wong P, Biggs SN (2016). Validation of actigraphy in middle childhood. Sleep.

[14] Leppänen MH, Migueles JH, Cadenas-Sanchez C (2020). Hip and wrist accelerometers showed consistent associations with fitness and fatness in children aged 8-12 years. Acta Paediatr.

[15] Yang X, Jago R, Zhang Q, Wang YY, Zhang J, Zhao WH (2019). Validity and reliability of the wristband activity monitor in free-living children aged 10-17 years. Biomed Environ Sci.

[16] Jean-Louis G, Kripke DF, Cole RJ (2001). Sleep detection with an accelerometer actigraph: comparisons with polysomnography. Physiol Behav.

[17] Quante M, Kaplan ER, Cailler M (2018). Actigraphy-based sleep estimation in adolescents and adults: a comparison with polysomnography using two scoring algorithms. Nat Sci Sleep.

[18] Boyne K, Sherry DD, Gallagher PR, Olsen M, Brooks LJ (2013). Accuracy of computer algorithms and the human eye in scoring actigraphy. Sleep Breath.

[19] Brindle RC, Yu L, Buysse DJ, Hall MH (2019). Empirical derivation of cutoff values for the sleep health metric and its relationship to cardiometabolic morbidity: results from the Midlife in the United States (MIDUS) study. Sleep.

[20] Smith C, Galland B, Taylor R, Meredith-Jones K (2019). Actigraph GT3X+ and actical wrist and hip worn accelerometers for sleep and wake indices in young children using an automated algorithm: validation with polysomnography. Front Psychiatry.

[21] Freedson P, Pober D, Janz KF (2005). Calibration of accelerometer output for children. Med Sci Sports Exerc.

[22] Butte NF, Wong WW, Lee JS, Adolph AL, Puyau MR, Zakeri IF (2014). Prediction of energy expenditure and physical activity in preschoolers. Med Sci Sports Exerc.

[23] Barreira TV, Schuna JM Jr, Mire EF, Katzmarzyk PT, Chaput JP, Leduc G, Tudor-Locke C (2015). Identifying children's nocturnal sleep using 24-h waist accelerometry. Med Sci Sports Exerc.

[24] Kinder JR, Lee KA, Thompson H, Hicks K, Topp K, Madsen KA (2012). Validation of a hip-worn accelerometer in measuring sleep time in children. J Pediatr Nurs.

[25] Reed DL, Sacco WP (2016). Measuring sleep efficiency: what should the denominator be?. J Clin Sleep Med.

[26] Coughlin SS (1990). Recall bias in epidemiologic studies. J Clin Epidemiol.

[27] Boulos MI, Jairam T, Kendzerska T (2019). Normal polysomnography parameters in healthy adults: a systematic review and meta-analysis. Lancet Respir Med.

[28] McLellan G, Arthur R, Donnelly S, Buchan DS (2020). Segmented sedentary time and physical activity patterns throughout the week from wrist-worn ActiGraph GT3X+ accelerometers among children 7-12 years old. J Sport Health Sci.

[29] Burns RD, Kim Y, Byun W, Brusseau T (2019). Associations of school day sedentary behavior and physical activity with gross motor skills: use of compositional data analysis. J Phys Act Health.

[30] Ranum BM, Wichstrøm L, Pallesen S, Falch-Madsen J, Halse M, Steinsbekk S (2019). Association between objectively measured sleep duration and symptoms of psychiatric disorders in middle childhood. JAMA Netw Open.

